# Editorial: Micro-nano optics and photocatalysis: inorganic material, heterojunction and mechanism insight

**DOI:** 10.3389/fchem.2024.1543435

**Published:** 2025-01-03

**Authors:** Shifa Wang, Qingping Ding, Angadi V. Jagadeesha, Hua Yang

**Affiliations:** ^1^ School of Electronic and Information Engineering, Chongqing Three Gorges University, Wanzhou, China; ^2^ Ames National Laboratory, U.S. Department of Energy, Iowa State University, Ames, IA, United States; ^3^ Department of Physics, P.C. Jabin Science College, Hubballi, Karnataka, India; ^4^ School of Science, Lanzhou University of Technology, Lanzhou, China

**Keywords:** micro-nano optical, optical devices, photocatalysts, heterojunction, solar cells

The key to the application of micro-nano optical and photocatalytic technology in the fields of optical devices, solar cells, laser devices memory devices, display devices, light emission devices, and photocatalysts is the development of new materials related to these technologies (Kumar et al., 2025; Jiménez-Calvo, 2024; Alli et al., 2024). The photocatalysts with special surface defects, heterojunction photocatalysts and metal particle surface decoration photocatalysts can be effectively synthesized by using advanced new material synthesis technology. The construction of heterojunction photocatalysts primarily involves type I, type II, Z-scheme, and S-scheme heterojunction photocatalysts (Han et al., 2024; Wang et al., 2025; Yu et al., 2024). A type I heterojunction is where the conduction and valence bands of one semiconductor are both inside the conduction and valence bands of another semiconductor. A type II heterojunction is one in which the conduction band or valence band of one semiconductor lies within the conduction band and valence band of another semiconductor. Z-scheme heterojunction refers to two or more kinds of semiconductors under the excitation of light, the conduction band (CB) and valence band (VB) of the corresponding semiconductor and the electrons and holes migrate to the corresponding other half of the conductor VB and CB respectively, so that the electrons and holes with strong reduction and oxidation ability of the two semiconductors can be maintained. The path of electrons and holes is similar to the letter Z. The S-scheme heterojunction is developed on the basis of the type II heterojunction, and its structure is an alternating arrangement of p-type semiconductor and n-type semiconductor, and the charge carrier transport mode is diffuse movement (Shabbir et al., 2024). The photocatalytic mechanisms of these heterojunction photocatalysts are obviously different due to the difference in charge transfer and separation efficiency. Therefore, how to develop new photocatalysts and construct multi-component heterojunction photocatalysts is the key to obtain efficient photocatalysts for degrading pollutants.

In this volume, the YMnO_3_/NiO photocatalyst was synthesized by one-step hydrothermal method. The photocatalyst for the degradation of oil and gas field wastewater has stable chemical structure and can be reused many times. Through the experiments of catalyst content, mass percentage of NiO and irradiation time, it was found that the best catalyst content of YMnO_3_/NiO photocatalyst was 1.5 g/L, the best mass percentage of NiO was 3%, and the best irradiation time was 60 min. The photocatalytic activity of the system is boosted by the effective transfer and separation of charge carriers by a type I band arranged heterojunction photocatalyst under the action of the internal electric field. This study provides a new technical reference for the degradation of oil and gas field wastewater.

Simultaneously, the application of surface modification technology in the field of photocatalysis can also greatly improve the photocatalytic activity of the photocatalyst (Wang et al., 2023). Generally, by modifying noble metal particles on the surface of semiconductor materials, the charge transfer and separation efficiency of semiconductor materials can be improved by using the surface plasmon resonance (SPR) effect of noble metal particles, so as to improve its photocatalytic activity. By grafting S-O bond on the surface of YMnO_3_, not only the photocatalytic activity of YMnO_3_ is enhanced, but also the YMnO_3_ can respond to ultraviolet to near-infrared light. This research contributes to the development of other novel near-infrared photocatalysts for the degradation of pollutants.

In addition, the optical properties of perovskite solar cells containing CH_3_NH_3_PbI_3_ were simulated and analyzed by a solar cell capacitance simulator. The acid-base process of titanium-containing blast furnace slag by concentrated sulfuric acid was simulated by establishing the kinetic model of acid hydrolysis. A geometric model of three-dimensional quarter-symmetric laser heating of quartz material was established by using nonlinear transient finite element method, which solved the problem that the phenomenon and physical mechanism under extreme conditions could not be explained experimentally. The transient temperature field distribution of quartz material after 1,064 nm continuous laser heating was studied. It can be seen that in the field of micro-nano optics and photocatalysis technology, it is particularly critical to properly use theoretical calculation to solve problems that cannot be solved by experiments.
